# Ventricular‐vascular coupling at rest and immediately following maximal exercise in adults with and without multiple sclerosis

**DOI:** 10.14814/phy2.71044

**Published:** 2026-08-12

**Authors:** Brooks A. Hibner, Natalia S. Lima, Sara R. Sherman, Robert W. Motl, Julio A. Chirinos, Shane Phillips, Philip S. Clifford, Anthony T. Reder, Tracy Baynard, Bo Fernhall

**Affiliations:** ^1^ Integrative Physiology Laboratory University of Illinois at Chicago Chicago Illinois USA; ^2^ Linda Crnic Institute for Down Syndrome University of Colorado Anschutz Medical Campus Aurora Colorado USA; ^3^ Hospital of the University of Pennsylvania Perelman School of Medicine at the University of Pennsylvania Philadelphia Pennsylvania USA; ^4^ Department of Physical Therapy University of Illinois at Chicago Chicago Illinois USA; ^5^ Department of Neurology University of Chicago Medicine Chicago Illinois USA; ^6^ Department of Exercise and Health Sciences University of Massachusetts Boston Boston Massachusetts USA

**Keywords:** cardiovascular function, exercise recovery, multiple sclerosis, ventricular‐vascular coupling

## Abstract

Multiple sclerosis (MS) may impair ventricular‐vascular coupling (VVC), a measure of the interaction between the cardiac and vascular systems. We evaluated if VVC differed between individuals with and without MS with similar age‐predicted fitness. Individuals with MS (*n* = 20) and without (CON, *n* = 18) underwent VVC measurement before and immediately after a maximal incremental cycle test with peak oxygen consumption (VO_2_peak) measurement. VVC was quantified as the ratio of arterial elastance (E_A_: end‐systolic pressure (ESP)/stroke volume) to ventricular elastance (E_LV_: ESP/end‐systolic volume). ESP was assessed with radial applanation tonometry, and cardiac volumes were obtained from apical four‐chamber echocardiography. Sex, BMI and VO_2_peak were not different between groups. Individuals with MS were older, had higher E_A_ (*p* < 0.01) and E_LV_ (*p* = 0.02), but VVC did not differ between groups. After adjustment for age, the group differences in E_A_ and E_LV_ remained significant. VVC was similar between individuals with and without MS, before and immediately after maximal exercise. However, individuals with MS had higher E_A_ and E_LV_, suggesting higher arterial and ventricular loads. Our data suggest that individuals with MS with similar fitness to controls exhibit higher E_A_ and E_LV_ with preserved coupling.

## 1 INTRODUCTION

Multiple sclerosis (MS) is a chronic neuroinflammatory disease of the central nervous system and a leading cause of nontraumatic disability in young adults (Dendrou et al., 2015; Reich et al., 2018; Wallin, Culpepper, Campbell, et al., 2019). The prevalence of MS is increasing and despite advances in disease‐modifying therapies, there is no cure (Goodin et al., 2012; Wallin, Culpepper, Campbell, et al., 2019). MS affects individuals throughout their adult life and is associated with an increased risk of cardiovascular disease, as individuals with MS have nearly twice the risk of cardiovascular mortality compared to those without the disease (Marrie et al., 2015; Palladino et al., 2020; Persson et al., 2020).

Exercise capacity, a strong predictor of cardiovascular disease mortality in the general population, is typically lower in individuals with MS (Langeskov‐Christensen et al., 2015; Mora et al., 2003). In those with MS, lower exercise capacity is related to disease progression, impaired mobility, reduced quality of life, and decreased independence (Motl & Pilutti, 2012). Exercise capacity depends not only on the cardiac and vascular systems, but also on their interaction. Ventricular‐vascular coupling describes the relationship between ventricular contractility and arterial load, reflecting the efficiency with which the ventricle generates pressure against the arterial load and serves as an integrated measure of cardiovascular performance (Chirinos, 2012, 2022; Ikonomidis et al., 2019; Kass, 2005). Ventricular‐vascular coupling is traditionally assessed as the ratio of arterial elastance (E_A_), an index of arterial load imposed on the heart, to ventricular elastance (E_LV_), an index of ventricular contractility (Chantler, 2017; Chirinos, 2012; Sunagawa et al., 1983). Elastance represents the change in pressure required for a change in volume, with higher values representing greater pressure required for a given change in volume (Chirinos, 2012; Sunagawa et al., 1983).

At rest, an ideal cardiovascular system optimizes efficiency with matched arterial and ventricular elastance, resulting in a coupling ratio of about one, which favors efficient energy transfer from the ventricle to the arterial system, rather than maximal stroke work (Chirinos, 2012). During exercise, cardiovascular demand increases and efficiency is sacrificed to prioritize greater work, resulting in a reduced coupling ratio during exercise (Asanoi et al., 1990; Currie et al., 2019; Najjar et al., 2004). The reduction in coupling is driven by a greater relative increase in ventricular elastance (ventricular contractility) compared to arterial elastance (arterial load) (Asanoi et al., 1990; Najjar et al., 2004). Immediately post exercise, coupling remains lower than rest. However, unlike during exercise, the reduction is driven by decreases in arterial elastance, with continued elevated ventricular elastance (Cote et al., 2013; Fahs et al., 2013).

Recovery from exercise is more than a passive return to rest; it is a dynamic phase marked by significant physiological changes (Romero et al., 2017). Impaired recovery, specifically blunted heart rate recovery post maximal exercise, is a strong, independent predictor of mortality, highlighting the importance of understanding cardiovascular performance during the immediate post exercise period (Cole et al., 1999). In the general population, higher ventricular‐vascular coupling is related to lower exercise capacity and is also independently associated with increased cardiovascular mortality (Fahs et al., 2013; Milewska et al., 2016; Wong et al., 2010).

Individuals with MS exhibit altered ventricular and vascular function compared to controls (Mincu et al., 2018; Ranadive et al., 2012). Because ventricular‐vascular coupling reflects the interaction between these systems, coupling may also be altered in this population. Given the blunted exercise responses and elevated cardiovascular mortality risk in individuals with MS, assessing ventricular‐vascular coupling at rest and during the immediate post exercise recovery period may provide important insight into cardiovascular performance in this population. However, ventricular‐vascular coupling has not been described in individuals with MS.

Therefore, our objective was to evaluate ventricular‐vascular coupling at rest and immediately after exercise in individuals with and without MS who have similar age‐predicted aerobic capacities. We aimed to assess the potential role of ventricular‐vascular coupling as a contributor to low exercise capacity and elevated cardiovascular risk in individuals with MS.

## 2 METHODS

### 2.1 Participants

Twenty individuals with MS and eighteen individuals without MS were included in this study. Individuals with MS included in this study were stable, free from episodic neurologic relapses for at least 30 days, and without change in disease modifying therapy for at least 6 months. More than half (*n* = 11/20) of participants reported exercise of at least 30 min on two or more days per week. Disease diagnosis and exercise clearance was confirmed by the participant's neurologist. Only individuals with relapsing–remitting MS and those able to complete cycle ergometry were enrolled. Disability was assessed using the Kurtzke's Expanded Disability Status Scale (EDSS), by a trained and certified assessor (Neurostatus level C) (Goldman et al., 2010; Rudick et al., 2010). Individuals with an EDSS of six or less were included as they would most likely be able to complete cycle ergometry, while still allowing a wide range of disability.

All participants recruited for this study had no history of cardiac or pulmonary disease, were not pregnant or current smokers, and had a body mass index (BMI) < 40 kg/m^2^. Each participant was fully informed of the purpose of the study, provided written informed consent for the protocol approved by the University of Illinois Chicago Institutional Review Board (2022–0177). All procedures were conducted in accordance with the ethical standards of the institutional research committee and the Declaration of Helsinki. Recruitment occurred through local MS support groups, community advertisement, ResearchMatch, word of mouth, and Chicago‐based neurology clinics.

### 2.2 Study design

Participants arrived at the Integrative Physiology Laboratory postprandial (≥3 h) and free from caffeine for at least 24 h. After informed consent, health history questionnaire, EDSS assessment (if applicable), weight (kg), and height (cm) were recorded. Following anthropometric measures, participants moved to a quiet, dimly lit room and after 10 min of supine rest, underwent baseline blood pressure (BP), heart rate (HR), and ventricular‐vascular coupling measurements, as described below. Participants then underwent a maximal cardiopulmonary exercise test on an upright cycle ergometer to measure exercise capacity as peak oxygen consumption (peak VO_2_), also described below. Immediately after maximal exercise, participants moved from the cycle ergometer to the supine position for BP, HR, and ventricular‐vascular coupling measurements. All measurements were obtained simultaneously and completed within 6 min of maximal exercise completion.

### 2.3 Blood pressure

Baseline and immediate postexercise BP were measured in the right arm at heart level in duplicate via an automated oscillometric cuff (HEM‐907 XL, Omron Healthcare, Lake Forest, IL). If duplicate BP values were not within 5 mmHg of each other, a third measurement was taken. Duplicate values were averaged and used for analysis. Mean arterial blood pressure (MAP) was calculated as $\mathrm{MAP}=\mathrm{DBP}+\frac{1}{3}\left(\mathrm{SBP}-\mathrm{DBP}\right)$.

### 2.4 Cardiac measurements

B‐mode Doppler echocardiography was performed with high‐fidelity ultrasound (Prosound Alpha 7, Hitachi‐Aloaka; Tokyo, Japan) and 2.5–5.0 MHz Doppler probe. Volume measurements of the left ventricle were obtained using the four‐chamber apical view and estimated with the biplane disk summation method (Mitchell et al., 2019). Images of 3–5 heart beats were traced to obtain average volumes. Stroke volume (SV) was calculated by subtracting end‐systolic volume (ESV) from end‐diastolic volume (EDV).

### 2.5 Vascular measurements

Radial pressure waveforms were acquired via applanation tonometry and averaged using 10 s epochs (SphygmoCor; AtCor Medical, Sydney Australia). A central aortic pressure waveform was reconstructed using a generalized validated transfer function to determine left ventricle end‐systolic pressure (ESP) (Sharman et al., 2006). Radial pressure waveforms were calibrated to brachial MAP and brachial diastolic blood pressure. This technique has been validated and is reliable (Holland et al., 2008; Sharman et al., 2006).

### 2.6 Ventricular‐vascular coupling (VVC) measurements

Ventricular‐vascular coupling was assessed via the relationship of pressure to volume, or elastance. Ventricular‐vascular coupling is calculated as the ratio of arterial elastance (E_A_) to ventricular elastance (E_LV_). such that 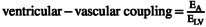, with $E_{A}=\frac{\mathrm{ESP}}{\mathrm{SV}}$, and $E_{\mathrm{LV}}=\frac{\mathrm{ESP}}{\mathrm{ESV}}$.

### 2.7 Aerobic capacity

Peak aerobic capacity, measured as peak oxygen consumption (peak VO_2_), was assessed using a maximal incremental exercise test on an upright cycle ergometer, with an open‐circuit spirometry system to analyze expired gases (TrueOne, Parvo Medics, Sandy, UT). The exercise protocol used for all participants was designed for persons with MS (Klaren et al., 2016; Motl & Fernhall, 2012). Briefly, participants began with a 3‐min warm‐up at 0 watts, followed by a 15‐watt per minute load increase until volitional exhaustion (defined as being unable to maintain 60 revolutions per minute on the cycle ergometer, despite encouragement). Peak effort was determined using standardized criteria, defined as satisfying two of the three following criteria: respiratory exchange ratio ≥1.10; peak heart rate within 10 beats per minute of age‐predicted maximum; or peak rating of perceived exertion (RPE) ≥17 (Klaren et al., 2016; Motl & Fernhall, 2012). Peak VO_2_ was averaged using 30 s epochs, using the highest recorded value. HR was recorded via Polar v800 heart rate monitor (Polar Electro Oy, Kempele, Finland), BP was measured via standard sphygmomanometry, and RPE was obtained at baseline and throughout the test. Exercise testing followed the American College of Sports Medicine and American Heart Association guidelines (American College of Sports M, 2018; Balady et al., 2010; Myers et al., 2009; Rodgers et al., 2000).

### 2.8 Statistical analyses

Data are presented as mean ± standard deviation. Data normality and outliers were checked via Shapiro–Wilk test. Nonnormality was corrected using the Log_10_ or reciprocal transformation. Frequency distribution of men and women in each group was tested using Chi‐Square. Independent *t*‐tests were used to examine potential age, BMI, and peak VO_2_ differences between groups (MS and CON). Bivariate correlation analysis was used to examine the relationship between ventricular‐vascular coupling and peak VO_2_. A group (MS, CON) by time (baseline, immediately post exercise) repeated measure analysis of variance (ANOVA) was used to evaluate ventricular‐vascular coupling responses, which included E_A_, E_LV_, Coupling, ESP, SV, and ESV. To account for the age difference between groups, age adjusted group by time repeated measures analysis of covariance (ANCOVA) was also performed. A priori alpha level of *p* ≤ 0.05 was deemed statistically significant and all analyses were performed using Statistical Package for the Social Sciences (SPSS, version 28, IBM, Chicago, IL, USA).

## 3 RESULTS

Descriptive characteristics of the participants are presented in Table 1. The MS group was primarily female (80%), which is consistent with population trends (Wallin, Culpepper, Nichols, et al., 2019; Wallin, Culpepper, Campbell, et al., 2019). Peak VO_2_, BMI, and sex did not differ between groups, although individuals with MS were older than controls. As peak VO_2_ was similar despite age differences, the percentage of age‐predicted peak VO_2_ was significantly higher in the MS group. Disease‐modifying therapies are also included in Table 1. Participants were not taking any additional comorbid drug therapy; those on drugs affecting the chronotropic or inotropic state of the heart were excluded.

**TABLE 1:** Descriptive characteristics of Individuals with and without MS.

	MS	Control	*p* Value
Female/Male	16/4	13/5	0.57
Age (years)	43 ± 10	29 ± 6	0.03
BMI (kg/m^2^)	25.5 ± 5.0	25.3 ± 4.4	0.59
EDSS^a^	3.5 (2.5–5.0)	—	—
Peak VO_2_ (mL/kg^−1^/min^−1^)	28.9 ± 8.0	30.7 ± 7.9	0.98
Age Predicted Peak VO_2_	30.8 ± 6.7	36.5 ± 7.5	0.52
Percent of Age Predicted Peak VO_2_	95 ± 22	84 ± 12	0.05
Disease Modifying Therapy (Trade name)			
Ocrelizumab (Ocrevus)	5	—	
Natalizumab (Tysabri)	3	—	
Ofatumumab (Kesimpta)	3	—	
Fingolimod (Gylenia)^b^	2	—	
Dimethyl fumarate (Tecfidera)	1	—	
Diroximel fumarate (Vumerity)	1	—	
Glatiramer acetate (Copaxone)	1	—	
None	4	—	

*Note*: Data presented as mean ± standard deviation.

Abbreviations: BMI, Body Mass Index; EDSS, Expanded Disability Status Scale; Peak VO_2_, Peak Oxygen uptake.

^a^ Presented as median (IQR).

^b^ Fingolimod is associated with an increased risk of cardiovascular events.

One MS participant did not have baseline and post maximal exercise ventricular‐vascular coupling measurements, therefore 19 individuals with MS and 18 without are included in the analysis. Arterial elastance (E_A_) and ventricular elastance (E_LV_) were higher in individuals with MS (*p* < 0.05) but ventricular‐vascular coupling was not different between groups (*p* = 0.41).

**FIGURE 1:**
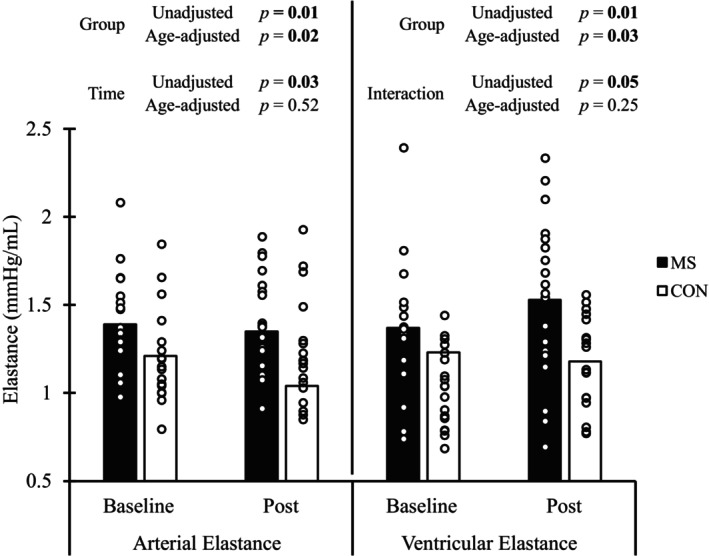
Arterial and Ventricular Elastance at Baseline and Immediately Post Peak Exercise in Individuals with and without MS.

**FIGURE 2:**
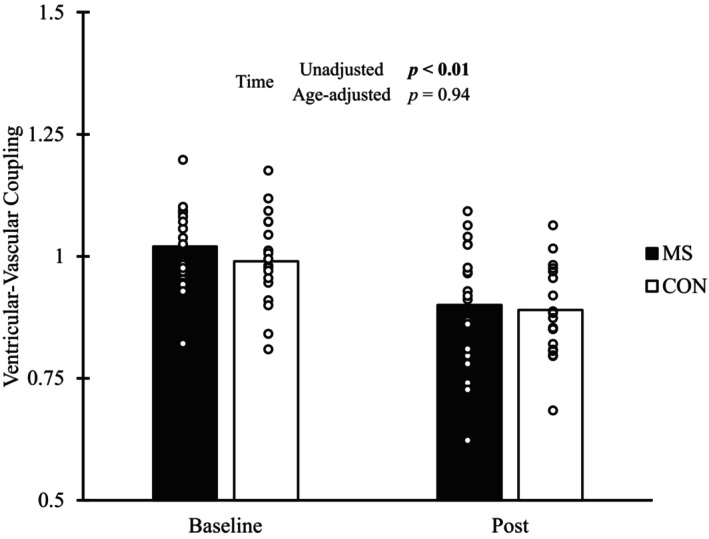
Ventricular‐Vascular Coupling at Baseline and Immediately Post Peak Exercise in Individuals with and without MS.

Arterial elastance decreased from baseline to immediate post maximal exercise (Figure 1, Pooled baseline‐immediate post exercise means ± S.D.; E_A_: 1.30 ± 0.27 mmHg/mL to 1.19 ± 0.31 mmHg/mL, *p* = 0.03) and was higher in individuals with MS (Figure 1, Pooled baseline‐immediate post exercise means ± S.D.; MS E_A_: 1.37 ± 0.26 mmHg/mL vs. CON E_A_: 1.21 ± 0.25 mmHg/mL, *p* = 0.01). After adjustment for age, E_A_ group difference remained significant (*p* = 0.02), while the time effect was no longer significant (*p* = 0.52). There was a significant time*group interaction for E_LV_, as individuals with MS exhibited a greater change in E_LV_ from pre to post maximal exercise (Figure 1, *p* = 0.05). Although the overall group difference in E_LV_ remained significant after age adjustment (*p* = 0.03), the E_LV_ group*time interaction was no longer significant. Ventricular‐vascular coupling decreased from baseline to immediate post exercise (Figure 2, Pooled baseline‐immediate post exercise means ± S.D.; VVC: 1.01 ± 0.12 to 0.89 ± 0.12, *p* < 0.01). There was no main group effect for ventricular‐vascular coupling (Figure 2, *p* = 0.27), which remained unchanged after adjustment for age (*p* = 0.86) However, the time effect for coupling was no longer significant following age adjustment (*p* = 0.94). Baseline ventricular‐vascular coupling was inversely related to exercise capacity (r = −0.45, *p* < 0.01).

Baseline and immediate post maximal exercise responses for ventricular‐vascular coupling components (ESP, SV, ESV), along with heart rate and mean arterial pressure, are presented in Table 2. ESP and ESV both decreased from baseline to immediate post maximal exercise (Pooled baseline‐immediate post exercise means ± S.D.; ESP: 89.9 ± 7.4 mmHg to 83.2 ± 12.2 mmHg, *p* < 0.01; ESV: 72.5 ± 17.4 mL to 65.4 ± 17.9 mL, p < 0.01) while SV did not change (*p* = 0.68). Individuals with MS had significantly higher ESP (Pooled baseline‐immediate post exercise means ± S.D.; MS ESP: 91.0 ± 8.1 mmHg vs. CON ESP: 82.0 ± 8.1 mmHg, *p* < 0.01), but there were no main group effects for SV or ESV (*p* = 0.18 and *p* = 0.34, respectively). There was no group*time interaction effect for ESP, SV, or ESV. Following adjustment for age, the time effect for ESP remained significant (*p* = 0.03), while the group effect for ESP and time effect for ESV was no longer significant (*p* = 0.19 and *p* = 0.28, respectively).

**TABLE 2:** Hemodynamic components at baseline and immediate postexercise in individuals with and without MS.

		Baseline	Post	Time		Group		Interaction	
				Raw	Age‐adjusted	Raw	Age‐adjusted	Raw	Age‐adjusted
ESP	MS	92.8 ± 7.3	89.2 ± 12.4	<0.01	0.03	<0.01	0.19	0.13	0.87
	CON	86.9 ± 7.5	77.1 ± 12.0						
SV^a^	MS	69 ± 14	69 ± 15	0.68	0.35	0.18	0.09	0.66	0.31
	CON	75 ± 18	77 ± 19						
ESV^a^	MS	70.4 ± 15.8	62.8 ± 19.2	<0.01	0.28	0.34	0.12	0.51	0.51
	CON	74.7 ± 18.9	68.0 ± 16.5						
HR	MS	65 ± 15	95 ± 17	0.01	0.01	0.99	0.49	0.44	0.20
	CON	63 ± 9	96 ± 10						
MAP	MS	85 ± 7	86 ± 8	0.25	0.13	<0.01	0.02	0.02	0.32
	CON	79 ± 6	75 ± 9						

*Note*: Data presented as mean ± standard deviation.

Abbreviations: ESP, End‐Systolic Pressure (mmHg); ESV, End‐Systolic Volume (mL); HR, Heart Rate (beats/min); MAP, Mean Arterial Pressure (mmHg); SV, Stroke Volume (mL).

^a^ Log_10_ transformed. Raw *p*‐values are from repeated‐measures ANOVA. Age‐adjusted *p*‐values are from repeated‐measures ANCOVA with age included as a covariate.

HR increased from baseline to post exercise (Pooled baseline‐immediate post exercise means ± S.D.; HR: 64 ± 9 bpm to 95 ± 10 bpm, *p* < 0.01) and remained significant after adjustment for age (*p* = 0.01) but was not different between groups (*p* = 0.44; age adjusted *p* = 0.20). There was no difference between baseline and post exercise MAP (*p* = 0.25). There was significant time*group interaction driven by a reduction in MAP following maximal exercise in the controls, whereas the group with MS did not change (p < 0.01). Following adjustment for age, the MAP interaction was no longer significant (p = 0.32), however, the group differences in MAP remained significant (*p* = 0.02).

## 4 DISCUSSION

Individuals with MS have an increased risk of cardiovascular mortality and typically have low exercise capacity, which could be related to impaired ventricular‐vascular coupling (Chantler, 2017; Klaren et al., 2016; Langeskov‐Christensen et al., 2015). However, ventricular‐vascular coupling has not been previously investigated in this population. This study investigated ventricular‐vascular coupling in individuals with MS before and immediately after maximal exercise. Our data support an inverse relationship between fitness and ventricular‐vascular coupling, consistent with findings in the general population (Fahs et al., 2013). Furthermore, although coupling before and immediately following peak exercise was similar between individuals with and without MS, both arterial and ventricular elastance were greater in individuals with MS. Importantly, these differences remained significant after adjustment for age. When unadjusted for age, arterial elastance decreased in both groups and people with MS exhibited a greater change in ventricular elastance immediately post peak exercise. However, after age adjustment, neither the arterial elastance time effect nor the ventricular elastance interaction remained significant. These findings suggest that the differences in elastance (arterial and ventricular) between those with MS and controls were not explained by age, but the age differences, rather than having MS, may have contributed to the immediate post exercise response. Therefore, while overall coupling was maintained and not different between groups, individuals with MS required higher arterial and ventricular elastances than controls to maintain coupling.

Earlier studies have shown that individuals with MS have low exercise capacity, partly related to disease‐mediated physical disability (Klaren et al., 2016; Motl & Pilutti, 2012). However, while our cohort included a wide range of physical disability, the group was exceptionally fit compared to prior studies. This group of individuals with MS achieved more than 90% of the general population's age predicted peak VO_2_ (95% ± 22%. min: 57%, max: 147%) (de Souza e Silva et al., 2018; Myers et al., 2021). While low peak VO_2_ has been documented in this population, on average compared to other studies with similar ages, this cohort's exercise capacity was about 30% greater than average (Hibner et al., 2020; Klaren et al., 2016; Langeskov‐Christensen et al., 2014; Motl & Fernhall, 2012). In a large meta‐analysis of 1029 individuals with MS that included a wide age and disability range, the average peak VO_2_ was 25.5 mL·kg^−1^·min^−1^, considerably lower than our cohort's peak VO_2_ of 28.9 mL·kg^−1^·min^−1^, representing a 13% difference (Langeskov‐Christensen et al., 2015). The relatively high fitness of our cohort may have contributed to the preserved coupling that we observed, despite individuals with MS exhibiting greater arterial and ventricular elastances.

Although both those with and without MS maintained a coupled system, it is important to note those with and without MS achieved coupling differently. Coupling is defined by the ratio of arterial to ventricular elastance; thus, to maintain coupling, changes in arterial elastance must be met by the same changes in ventricular elastance. At rest, individuals with MS exhibited a higher arterial elastance compared to controls. Thus, to maintain coupling, higher arterial elastance was matched with higher ventricular elastance in individuals with MS. While individuals in our study matched higher arterial elastance with greater ventricular elastance and maintained a coupled system, this may suggest that although the system is coupled, it may not be optimal.

Individuals with MS exhibited higher arterial elastance than controls, suggesting an increased arterial load imposed on the heart. Arterial load is a complex hemodynamic phenomenon including timing, resistance, compliance, and impedance which together characterize the external factors that oppose ventricular ejection (Segers et al., 2002; Vlachopoulos et al., 2011). Arterial elastance lumps those properties together to serve as an index of arterial load, or total load imposed on the heart (Chantler, 2017; Chirinos, 2012). Individuals with MS exhibited higher arterial elastance than controls, suggesting a greater arterial load, and thus a greater workload on the heart. Arterial elastance is characterized as the ratio of end‐systolic pressure to stroke volume; higher arterial elastance can result from higher end‐systolic pressure, lower stroke volume, or both. In our cohort, stroke volume was not different, but individuals with MS had higher end‐systolic pressures. However, once adjusted for age, the group difference in end‐systolic pressure was no longer significant. Thus, age may contribute to the difference in end‐systolic pressure in our groups. Despite this, arterial elastance remained significantly higher in those with MS after age adjustment. Therefore, the higher arterial elastance in our MS cohort is not fully explained by age differences in end‐systolic pressure; instead, it may be reflective of a combination of hemodynamic factors. Given arterial elastance is the ratio of ESP to stroke volume, a higher arterial elastance indicates that greater pressure is required to achieve a given stroke volume (Kass, 2002). The resultant increased pressure increases myocardial oxygen consumption and thus work for a given stroke volume (Chirinos, 2012; Kawaguchi et al., 2003; Kelly et al., 1992). Consequently, these findings suggest that individuals with MS may experience increased arterial load and result in greater workload of the system despite maintaining a similar stroke volume as controls.

Higher arterial elastance was matched with higher ventricular elastance in individuals with MS. Ventricular elastance serves as a marker of contractility and ventricular stiffness (Chantler, 2017; Chirinos, 2012; Sunagawa et al., 1983). Higher resting ventricular elastance suggests an unfavorable energetic state and decreased contractile reserve, as further contractility increases have smaller effects on ejection (Chen et al., 1998; Kass, 2005; Kawaguchi et al., 2003). Ventricular elastance is the ratio of end‐systolic pressure to end‐systolic volume and reflects the pressure generated for a given volume (Chantler, 2017; Kawaguchi et al., 2003). Thus, higher ventricular elastance indicates greater pressure generation for a given volume. In our cohort, there was no difference in ESV, even after adjustment for age. While individuals with MS exhibited higher end‐systolic pressure without age adjustment, once age was accounted for, the end‐systolic pressure differences were no longer significant. Again, like arterial elastance, ventricular elastance remained significantly elevated after age adjustment. Therefore, the higher ventricular elastance in those with MS cannot fully be explained by age differences in end‐systolic pressure and suggests a stiffer or more contractile ventricle in people with MS.

During exercise, coupling is expected to decrease due to greater relative change in ventricular elastance compared to arterial elastance (Chantler, 2017; Najjar et al., 2004). This is due to the significant cardiovascular demand of exercise, requiring increases in heart rate, stroke volume, and blood flow redistribution to active tissues (Lefferts et al., 2022). While the cardiovascular system at rest is coupled, prioritizing energetic efficiency, exercise shifts this balance in favor of greater cardiac work (Asanoi et al., 1990; Najjar et al., 2004). Both arterial elastance and ventricular elastance increase during exercise; however, ventricular elastance exhibits a greater relative increase due to greater increases in contractility and preload, resulting in a lower coupling ratio. Immediately post exercise, coupling remains lower compared to resting values (Cote et al., 2013; Fahs et al., 2013). However, the post exercise coupling is instead likely the result of postexercise vasodilation and decreased total peripheral resistance, reflected by a lower ESP (Fahs et al., 2013). In the unadjusted analysis, our findings are consistent with previous literature, showing reduced coupling in both groups immediately postexercise. This was accompanied by an increased ventricular elastance from baseline to immediately post exercise only in individuals with MS. However, after adjustment for age, neither the reduction in coupling nor the ventricular elastance interaction remained significant. This suggests age likely contributed to the immediate post exercise response.

One strength of this study was the methodology used to measure ventricular‐vascular coupling. We used arterial waveform analysis to obtain ESP through transformed radial pressure waveforms. This is superior to using estimated measures from standard brachial cuff measurements (Kappus et al., 2012). Although arterial and ventricular elastance are widely used to assess coupling, using elastance does simplify important properties of the cardiovascular system (Chantler & Lakatta, 2012; Chirinos, 2012; Sunagawa et al., 1983). Namely, arterial elastance is not sensitive to pulsatile changes and thus is not a pure index of total arterial load (Chirinos et al., 2014). Similarly, ventricular elastance is not likely purely a measure of cardiac load (Chirinos, 2012). Coupling can be assessed with more accurate measures, but they are more complicated, require additional expertise, and offer less intuitive interpretation. Another limitation is the relatively small sample size. However, to our knowledge, ventricular‐vascular coupling assessed via elastance has not previously been evaluated during exercise in individuals with MS; hence, we feel it is an important contribution to the literature that may serve future work in this area. Finally, in addition to control participants, our study only recruited individuals with relapsing–remitting MS who have EDSS scores 0–6, which were unexpectedly relatively fit. While our groups exhibited similar age‐predicted aerobic capacities, individuals with MS were older than controls. Given the effects of aging on ventricular‐vascular coupling, we performed additional analysis using age as a covariate. While the group differences in arterial and ventricular elastance remained significant after adjustment for age, the immediate postexercise responses did not. Although age differences do not fully explain the higher elastance (arterial and ventricular), the difference in age may have contributed to some of the observed immediate postexercise responses. Therefore, although the primary group differences in arterial and ventricular elastance remained significant after adjustment for age, our findings are only applicable to this specific group of relatively fit individuals with relapsing–remitting MS and do not apply to other forms of MS or to individuals with an EDSS greater than six.

## 5 CONCLUSION

In conclusion, we found that ventricular‐vascular coupling was similar between individuals with and without MS, both before and immediately after maximal exercise. While coupling was maintained in both groups, individuals with MS exhibited higher arterial and ventricular elastances at baseline and post exercise. Importantly, these differences remained significant after adjustment for age, suggesting they were not simply due to the older age of the MS group. Collectively, these findings suggest that individuals with MS experience greater cardiovascular workload and reduced efficiency despite maintaining ventricular‐vascular coupling. Considering the individuals with MS in this study were relatively fit, the ability to match higher arterial elastances with increased ventricular elastance was not limited; however, this compensation may not be possible in a more typical, lower fit group of individuals with MS. These findings provide new insight into the interaction between ventricular and vascular function in individuals with MS. Nevertheless, coupling remained within normal range and likely did not limit cardiovascular performance.

## AUTHOR CONTRIBUTIONS

**Brooks A. Hibner:** Conceptualization; data curation; formal analysis; funding acquisition; investigation; methodology; project administration; validation; visualization. **Natalia S. Lima:** Investigation. **Sara R. Sherman:** Investigation. **Robert W. Motl:** Methodology; resources; supervision. **Julio A. Chirinos:** Conceptualization; methodology; supervision. **Shane Phillips:** Conceptualization; methodology. **Philip S. Clifford:** Conceptualization; methodology. **Anthony T. Reder:** Investigation; supervision. **Tracy Baynard:** Conceptualization; methodology; supervision. **Bo Fernhall:** Conceptualization; formal analysis; funding acquisition; methodology; resources; supervision.

## FUNDING

Dr. Chirinos is supported by National Institutes of Health grants U01‐TR003734, U01‐TR003734‐01S1, UO1‐HL160277, R33‐HL‐146390, R01‐HL153646, K24‐AG070459, R01‐AG058969, R01‐HL157108, R01‐HL155599, R01‐HL104106 and R01HL155764. He has recently consulted for Bayer, Fukuda‐Denshi, Bristol‐Myers Squibb, Johnson & Johnson, Edwards Life Sciences, Merck, and NGM Biopharmaceuticals. He received University of Pennsylvania research grants from National Institutes of Health, Fukuda‐Denshi, Bristol‐Myers Squibb, Microsoft, and Abbott. He is named as inventor in a University of Pennsylvania patent for the use of inorganic nitrates/nitrites for the treatment of Heart Failure and Preserved Ejection Fraction and for the use of biomarkers in heart failure with preserved ejection fraction. He has received payments for editorial roles from the American Heart Association, the American College of Cardiology, Elsevier, and Wiley, and payments for academic roles from the University of Texas, Boston University, and Virginia Commonwealth University. He has received research device loans from Atcor Medical, Fukuda‐Denshi, Unex, Uscom, NDD Medical Technologies, Microsoft, and MicroVision Medical. This project was supported by the Congressionally Directed Medical Research Program through the U.S. Army Medical Research and Material Command (W81XWH1810466) and the American College of Sports Medicine Foundation (21‐01556). The commercial entities providing research funding or research device loans had no role in the study design, data collection, data analysis, interpretation of the results, manuscript preparation, or the decision to submit the manuscript for publication.

## CONFLICT OF INTEREST STATEMENT

The results of the study are presented clearly, honestly, and without fabrication, falsification, or inappropriate data manipulation. The results of the present study do not constitute endorsement by the American College of Sports Medicine.

## ETHICS STATEMENT

The study protocol was approved by the University of Illinois Chicago Institutional Review Board (IRB Protocol #2022–0177). All participants provided written informed consent prior to participation. All procedures were conducted in accordance with the ethical standards of the institutional research committee and the Declaration of Helsinki.
